# Chloroquine Ingestion to Prevent SARS-CoV-2 Infection: A Report of Two Cases

**DOI:** 10.5811/cpcem.2021.3.51329

**Published:** 2021-05-07

**Authors:** Jonathan Graff, Michelle Geyer Thompson, Zachary Berriochoa, Bryan Kuhn, Anne-Michelle Ruha, Christopher Lipinski

**Affiliations:** * Banner Baywood Medical Center, Department of Emergency Medicine, Mesa, Arizona; † Banner-University Medical Center Phoenix, Department of Medical Toxicology, Phoenix, Arizona; ‡ Banner Poison and Drug Information Center, Phoenix, Arizona; § University of Arizona College of Medicine-Phoenix, Departments of Emergency Medicine and Internal Medicine, Phoenix, Arizona; ¶ Midwestern University, Glendale, Arizona

**Keywords:** Chloroquine overdose, COVID-19, fish tank cleaner, SARS-CoV2, case report

## Abstract

**Introduction:**

Amid the global pandemic caused by severe acute respiratory syndrome coronavirus 2 (SARS-CoV-2), chloroquine and hydroxychloroquine were being studied as agents to prevent and treat coronavirus disease 2019. Information about these agents and their effects circulated throughout the general public media, raising the concern for self-directed consumption of both pharmaceutical and non-pharmaceutical products.

**Case Report:**

We present two cases of chloroquine toxicity that occurred after ingestion of an aquarium disinfectant that contained chloroquine phosphate in a misguided attempt to prevent infection by SARS-CoV-2. One patient had repeated emesis and survived, while the other was unable to vomit, despite attempts, and suffered fatal cardiac dysrhythmias.

**Conclusion:**

These cases illustrate the spectrum of toxicity, varied presentations, and importance of early recognition and management of chloroquine poisoning. In addition, we can see the importance of sound medical guidance in an era of social confusion compounded by the extremes of public and social media.

## INTRODUCTION

Chloroquine and its less toxic derivative, hydroxychloroquine, were first synthesized as antimalarial medications and received US Food and Drug Administration (FDA) approval in 1949 and 1955, respectively. Over the past 70 years, they have been repurposed as anti-inflammatory, antimicrobial, and immunomodulatory agents for rheumatologic syndromes, such as rheumatoid arthritis, Sjogren’s syndrome, lupus erythematosus, and various infections, such as extraluminal amoebiasis, Whipple’s disease, and Q fever.^1^–^3^ They may also have benefit as an antitumoral or in some neurological diseases.^4^ Early in the global coronavirus disease 2019 (COVID-19) pandemic, research emerged examining the efficacy of chloroquine and hydroxychloroquine against severe acute respiratory syndrome coronavirus 2 (SARS-CoV-2) replication.^5^,^6^

Commentary on these publications quickly spread across social media. The FDA temporarily provided emergency use authorization (EUA) on March 28, 2020, for possible management of SARS-CoV-2 and COVID-19, but revoked the EUA on June 15, 2020.^7^ Given the fast-paced dissemination of information from the medical community to the general public during the pandemic, it is critical for emergency physicians to recognize acute toxicity of chloroquine and implement early aggressive management.

Chloroquine has a very narrow therapeutic window, and is considered a “one pill can kill” exposure in the pediatric patient.^8^ Severe chloroquine toxicity is characterized by nausea, vomiting, diarrhea, seizures, abrupt decompensation due to cardiovascular collapse, and death within one to three hours of ingestion.^9^ Importantly, hypokalemia is a common finding and correlates with the severity of poisoning.^10^ Management must focus on early gastric decontamination including activated charcoal, timely hemodynamic support, mechanical ventilation, and electrolyte management. Fatal outcomes following overdose of chloroquine are associated with ingestions of greater than 5 grams (g), systolic blood pressure less than 80 millimeters of mercury (mm Hg), and QRS duration greater than 120 milliseconds (ms).^9^,^10^ In a very small study of 22 case reviews, where more than 5 g of chloroquine were ingested, the combination of preemptive mechanical ventilation, high-dose diazepam, and epinephrine infusion prior to the start of cardiac dysrhythmias appeared to increase the survival rate from 10% to 91%.^9^ Because of the risk of early fatality following toxic ingestions, it is imperative to quickly intervene following acute chloroquine poisoning.

**Image 1:**
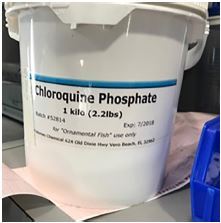
Chloroquine phosphate aquarium disinfectant.

We describe two cases of chloroquine toxicity that occurred simultaneously after a husband and wife each ingested a teaspoon of aquarium disinfectant containing 98% by weight chloroquine phosphate (Image 1) dissolved in carbonated water, believing it would help prevent infection by SARS-CoV-2.

## CASE 1

**Image 2:**
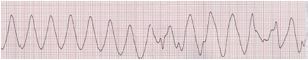
Wide complex ventricular tachycardia in patient who ingested chloroquine phosphate aquarium disinfectant.

The husband, a 68-year-old White man with a history of hypertension and dyslipidemia, developed diarrhea and nausea without emesis within 20 minutes of the ingestion. His wife called for medical assistance 90 minutes after the ingestion when he developed dyspnea. Paramedics found him to be alert, oriented, and diaphoretic with a blood pressure of 110/67 millimeters mercury (mm Hg), heart rate of 93 beats per minute, respiratory rate (RR) of 20 breaths per minute, and oxygen saturation of 90% on ambient air. His initial rhythm strip showed normal sinus rhythm. Paramedics administered sodium bicarbonate 100 milliequivalents (mEq) after communicating with the local poison control center, as well as 15 liters per minute (L/min) oxygen via non-rebreather mask and a normal saline (NS) bolus, amounting to about 300 mL. Upon arrival to the emergency department (ED), the patient became unresponsive, had a generalized tonic-clonic seizure, and developed a pulseless electrical activity (PEA) arrest. He was intubated, cardiopulmonary resuscitation (CPR) was initiated, and a total of epinephrine 3 milligrams (mg), sodium bicarbonate 50 mEq, atropine 2 mg and magnesium 1g were administered, along with continued NS infusion. The patient had recurrent episodes of wide complex ventricular tachycardia (Image 2).

**CPC-EM Capsule**

What do we already know about this clinical entity?

*Chloroquine has been widely used for decades with multiple applications. It has a narrow therapeutic window, is rapidly absorbed and has significant cardiac toxicities.*

What makes this presentation of disease reportable?

*The couple ingested the chloroquine in a misguided attempt to prevent SARS-CoV-2. We see their side-by-side response, and associated bidirectional tachycardia*

What is the major learning point?

*This pandemic and social media have compounded public fears and confusion. Sound local and regional medical leadership can help significantly with our society’s health.*

How might this improve emergency medicine practice?

*These cases can stimulate us to be more involved in our communities’ leadership and better prepared to intervene when minutes count.*

**Image 3:**
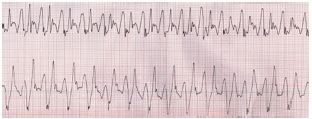
Bidirectional tachycardia in a patient who ingested chloroquine phosphate aquarium disinfectant.

**Image 4:**
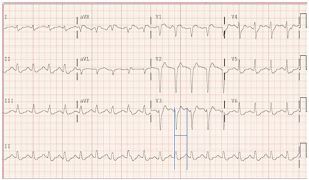
Sinus tachycardia and prolonged QTcFri interval 491 milliseconds in a patient who ingested chloroquine phosphate aquarium disinfectant.

In addition, an early rhythm showed bidirectional ventricular tachycardia (Image 3), which has not been previously reported with chloroquine toxicity. The patient received diazepam 10 mg intravenously (IV), which dose was readily available, and he had return of spontaneous circulation (ROSC) within two minutes (within 10 minutes of cardiac arrest). The electrocardiogram (ECG) (Image 4) showed sinus tachycardia with a rate of 111, QRS duration of 108 ms, and QTcFri (corrected QT interval using the Fridericia calculation considered the more accurate calculation, QTc = 8.22 cube root of RR, where RR is the pulse period) interval prolonged to 491 ms. Seven minutes later, the patient had another PEA arrest with subsequent CPR.

He received epinephrine 4 mg, sodium bicarbonate 50 mEq and calcium chloride 1g in total, and ROSC was achieved 11 minutes later. Epinephrine and norepinephrine infusions were started to augment blood pressure. A third and final PEA arrest occurred nine minutes after ROSC, and again CPR was performed with a total administration of epinephrine 9 mg, diazepam 10 mg, sodium bicarbonate 50 mEq, atropine 1mg, and magnesium sulfate 3g. Resuscitation was terminated 27 minutes later without successful resuscitation. The patient was declared deceased three hours and 22 minutes after time of ingestion. The perimortem serum chloroquine concentration was 6.2 mg/L. Pharmacogenomic testing revealed normal metabolism status of cytochrome P(CYP)2D6 and CYP3A4 but intermediate metabolism of CYP3A5.

## CASE 2

**Image 5:**
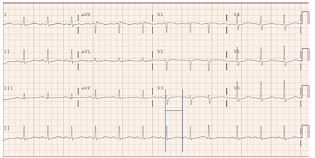
Prolonged QTcFri interval, 646 milliseconds in a patient who ingested chloroquine phosphate aquarium disinfectant.

The wife, a 62-year-old White female with a history of anxiety and insomnia developed nausea, recurrent emesis, mild diarrhea, and abdominal pain 25 minutes after the ingestion. She was transported to the local ED immediately after her husband where she reported dizziness, shakiness, and blurry vision. Initial vital signs showed a blood pressure of 104/72 mm Hg, heart rate of 73 beats per minute, RR of 17 breaths per minute, and room air oxygen saturation of 97%. Her initial ECG obtained 150 minutes post-ingestion showed sinus rhythm with a QRS duration of 108 ms and average QTcFri interval of 646 ms (Image 5). Labs revealed hypokalemia (3.3 millimoles [mmol] per L [reference range: 3.6–5.3 mmol/L). Sodium bicarbonate 50 mEq, ondansetron 4 mg, and magnesium sulfate 2 g were administered. She was transferred to a tertiary care hospital for further management. There, a repeat ECG obtained seven hours post-ingestion showed sinus rhythm of 79 beats/minute, with a QRS duration of 99 ms and QTcFri interval of 603 ms. She received a sodium bicarbonate infusion, lactated Ringer’s bolus, magnesium sulfate 2 g, lorazepam 0.5 mg and potassium chloride 20 mEq. Eleven hours post-ingestion her ECG showed a sinus rhythm 77 beats/minute, QRS 109 ms, and QTcFri of 560 ms. She complained of nausea, lightheadedness, and fatigue over the next 48 hours and had recurrent episodes of emesis and diarrhea. Sodium bicarbonate infusion was weaned without complication or widening of the QRS. Her symptoms of emesis resolved, and an ECG at the time of discharge showed a QRS duration of 90 ms and QTcFri interval of 428 ms. Serum chloroquine concentrations obtained three and 16 hours after ingestion were 1.1 mg/L and 0.4 mg/L, respectively. Pharmacogenomic testing revealed normal metabolism status of CYP2D6 and CYP3A4 but poor metabolism of CYP3A5.

## DISCUSSION

As demonstrated by these cases, chloroquine toxicity is difficult to manage and potentially fatal. Chloroquine is rapidly absorbed from the gastrointestinal tract, and serum concentrations peak within 1–2 hours of ingestion.^12^ Chloroquine is metabolized by various cytochrome P450 isoenzymes, with CYP2C8, CYP2D6 and CYP3A4/5 being the major catalysts of deethylation.^13^ The active metabolite, desethylchloroquine, can be detected in plasma within 30 minutes of an oral dose of chloroquine^12^ and can be found in serum for 30–60 days. Serum chloroquine concentrations greater than 5 mg/L are associated with fatality.^14^ This is consistent with the husband’s outcome. Due to the large volume of distribution of chloroquine, there is some thought that intralipid infusion may have benefit; however, this therapy has not been shown to be effective.^15^

The couple reported that they each consumed a single “heaping” teaspoon of chloroquine; however, the precise amount of ingested product is unknown. Since the wife developed recurrent emesis, she likely absorbed less chloroquine, had lower peak serum concentrations, and was at lower risk for developing cardiac dysrhythmias. This relationship is described in the literature and indicates that cardiac dysrhythmias are less likely if emesis occurs or gastric lavage is performed, even following ingestion of a fatal dose.^16^ Activated charcoal binds 95% of chloroquine when administered soon after ingestion and is recommended to supersede the use of gastric lavage due to the rapid absorption of chloroquine from the gastrointestinal tract.^17^ Importantly, both patients’ pharmacogenetic profiles showed similar variances in the function of CYP450 enzymes relevant to the metabolism of chloroquine. Although CYP2C8 status was not determined in either patient, it seems unlikely that pharmacogenetic differences explain the disparity in severity of illness and outcome.

In the second case, sodium bicarbonate was administered for the treatment of presumed sodium blockade. Mindful of the presence of hypokalemia and the risk of worsening both hypokalemia and QT prolongation with administration of sodium bicarbonate, potassium was aggressively replete. Upon transfer to the tertiary care hospital, the QRS had narrowed and signified a response to sodium bicarbonate administration.

Cardiotoxicity following chloroquine ingestion occurs as a result of inhibition of sodium, calcium, and potassium channels within the myocardium, leading to delayed depolarization, slowed conduction, prolonged refractory period, impaired contractility, reentrant dysrhythmias, and hypotension.^18^ In cases where more than 5 g of chloroquine are ingested, the combination of preemptive mechanical ventilation, high-dose diazepam (2 mg per kilogram [kg] IV over 30 minutes, followed by 2 mg/kg/day), and epinephrine infusion (0.25 micrograms/kg/minute to maintain a systolic blood pressure >100 mm Hg) during hemodynamic and electrocardiographic changes, has been a strategy in combating dysrhythmias and ultimately decreasing the risk of mortality.^9^,^10^

## CONCLUSION

During this time of heightened public fear and anxiety, information and misinformation about therapeutics for COVID-19 disease will continue to spread through the press and social media. It is important for public health officials and clinicians to effectively communicate the limitations and dangers of using unproven therapies to prevent or treat SARS-CoV-2 infection. Additionally, the cases presented serve as a critical reminder that emergency physicians must be prepared to quickly recognize, assess, and treat toxicities from acute poisoning by chloroquine and hydroxychloroquine.

## References

[1] Rolain JM, Colson P, Raoult D (2007). Recycling of chloroquine and its hydroxyl analogue to face bacterial, fungal and viral infections in the 21st century. Int J Antimicrob Agents.

[2] Savarino A, Boelaert JR, Cassone A (2003). Effects of chloroquine on viral infections: an old drug against today’s diseases. Lancet Infect Dis.

[3] Vincent MJ, Bergeron E, Benjannet S (2005). Chloroquine is a potent inhibitor of SARS coronavirus infection and spread. Virol J.

[4] Plantone D, Koudriavtseva T (2018). Current and future use of chloroquine and hydroxychloroquine in infectious, immune, neoplastic, and neurological diseases: a mini-review. Clin Drug Investig.

[5] Liu J, Cao R, Xu M (2020). Hydroxychloroquine, a less toxic derivative of chloroquine, is effective in inhibiting SARS-CoV-2 infection in vitro. Cell Discov.

[6] Gautret P, Lagier JC, Parola P (2020). Hydroxychloroquine and azithromycin as a treatment of COVID-19: results of an open-label non-randomized clinical trial. Int J Antimicrob Agents.

[7] Federal Drug Administration (2020). Emergency Use Authorization of chloroquine phosphate and hydroxychloriquine sulfate.

[8] Koren G (1993). Medications which can kill a toddler with one tablet or teaspoonful. J Toxicol Clin Toxicol.

[9] Riou B, Barriot P, Rimailho A (1988). Treatment of severe chloroquine poisoning. New Engl J Med.

[10] Riou B, Rimailho A, Galliot M (1988). Protective cardiovascular effects of diazepam in experimental acute chloroquine poisoning. Intensive Care Med.

[11] Clemessy JL, Taboulet P, Hoffman JR (1996). Treatment of acute chloroquine poisoning: a 5-year experience. Crit Care Med.

[12] Walker O, Dawodu A, Adeyokunnu A (1983). Plasma chloroquine and desethylchloroquine concentrations in children during and after chloroquine treatment for malaria. Br Journal Clin Pharmacol.

[13] Projean D, Baune B, Farinotti R (2003). In vitro metabolism of chloroquine: identification of CYP2C8, CYP3A4, and CYP2D6 as the main isoforms catalyzing N-desethylchloroquine formation. Drug Metab Dispo.

[14] Stead A, Moffat A (1983). A collection of therapeutic, toxic and fatal blood drug concentrations in man. Human Exp Toxicol.

[15] Haesendonck R, Winter S, de Verelst S (2012). Intravenous lipid emulsion for intentional chloroquine poisoning. Clin Toxicol (Phila).

[16] Britton W, Kevau I (1978). Intentional chloroquine overdosage. Med Journal Aust.

[17] Kivistö KT, Neuvonen PJ (1993). Activated charcoal for chloroquine poisoning. BMJ.

[18] White NJ (2007). Cardiotoxicity of antimalarial drugs. Lancet Infect Dis.

